# Exploring the Effect of Selenidation Time on the Ni-Doped Cu_2_ZnSn(S,Se)_4_ Solar Cell

**DOI:** 10.3390/nano12234311

**Published:** 2022-12-05

**Authors:** Fancong Zeng, Jingshu Wang, Meiling Ma, Na Zhao, Tianyue Wang, Guangbo Chen, Bin Yao, Yingrui Sui

**Affiliations:** 1 Key Laboratory of Functional Materials Physics and Chemistry of Ministry of Education, Jilin Normal University, Changchun 130103, China; 2 State Key Laboratory of Superhard Materials and College of Physics, Jilin University, Changchun 130012, China

**Keywords:** CNZTSSe, thin film, properties, solar cells, selenidation time

## Abstract

The Cu_2_Ni_0.05_Zn_0.95_Sn(S,Se)_4_ (CNZTSSe) films were synthesized by sol-gel combined with selenidation treatment. To further enhance the crystal quality of the film, the selenidation conditions were optimized, and the effects of selenidation time on the properties of the CNZTSSe films and devices were systematically studied. The results show that the crystallinity of the films increased remarkably with the increase of selenidation time. Under the optimum selenidation time of 15 min, smooth and dense films were obtained. Through the analysis of EDS results, it is found that Se occupies more S positions with the increase of selenidation time, which decreases the band gap of the film from 1.14 eV to 1.0 eV. In addition, the formation of Zn-related defects is effectively suppressed by Ni doping to enhance the open circuit voltage (V_oc_) of the CNZTSSe solar cells. When the selenidation time is 15 min, the CNZTSSe film has the highest carrier concentration of 1.68 × 10^16^ cm^−3^, and the best efficiency of the device prepared based on the film as the absorption layer is 5.0%, and the V_oc_ is 337 mV.

## 1. Introduction

Due to the high cost of silicon solar cells and the high requirements for the preparation process, photovoltaic cells based on thin film materials have emerged as the times require. There are mainly III or V groups of compound cells, such as gallium arsenide (GaAs), cadmium telluride (CdTe), copper indium selenium (CIS), copper indium gallium selenium (CIGS). CIGS thin film solar cells are the best of the second generation solar cells [1]. At the end of 2015, the German Renewable Energy Laboratory (ZSW) announced the preparation of CIGS thin film solar cells with an efficiency of 21.7% [2]. However, it contains rare elements such as indium (In) and gallium (Ga). Because of its low reserves and rising prices year by year, the cost of CIGS solar cells has greatly increased, severely limiting their large-scale application in practice. Therefore, the third-generation Cu_2_ZnSn(S,Se)_4_ (CZTSSe) thin-film photovoltaic material with kesterite structure has become the best substitute for CIGS thin film due to its non-toxic, low preparation cost, good stability, and high absorption coefficient [3,4,5]. At the same time, the structure of CZTSSe thin film solar cells has borrowed from the commercial CIGS thin film solar cells [6]. To verify the photovoltaic effect of CZTSSe thin films, Nitsche and other researchers first studied the crystal growth of Cu_2_ZnSnS_4_ (CZTS) compounds using vapor diffusion [7]. Later, Kentaro and other researchers studied the electrical and optical properties of CZTS with stannite structure for the first time, thus experimentally confirming that this kind of film has a photovoltaic effect [8]. IBM Company of the United States used the hydrazine solution method to deposit CZTSSe film, which made the efficiency of the device reach 12.6% [9]. However, the toxicity and explosiveness of hydrazine may limit its commercialization. The recent exciting method is that Xin et al. made a breakthrough by using a simple solution method to make the efficiency of CZTSSe thin film solar cells reach 13% [10]. However, its efficiency is still far lower than commercial CIGS thin film solar cells [11]. According to relevant literature reports, it is found that low V_oc_ is the main bottleneck restricting the development of these solar cells [12].

The low V_oc_ is usually caused by poor crystal quality, many inherent defects in the crystal, and poor energy band matching. Many experiments and theoretical calculations indicate that the main inherent defect in CZTSSe crystal is Cu_Zn_ inversion [13,14]. Since the similar cation size of the two metal elements, high concentration Cu_Zn_ corrosion resistance defects (Cu_Zn_ disorder) will be formed, resulting in the emergence of band tail states, thus reducing the V_oc_ [15]. One way to alleviate Cu_Zn_ disorder is to make the absorber undergo disorder order transition by low-temperature annealing. However, the relevant literature shows that this method needs to be combined with high-temperature post-annealing, and the promotion effect on V_oc_ is not obvious [16,17,18]. Another strategy to reduce Cu_Zn_ defects is to partially replace Cu or Zn with isoelectronic elements with different ion sizes to effectively improve the V_oc_ of the device, such as replacing copper with silver and zinc with cadmium [19,20,21]. However, the disadvantages of silver and cadmium substitution are high cost and toxicity. To avoid this problem, in our previous work, Ni atoms with non-toxic, abundant reserves, low cost, and close to the radius of zinc ions were introduced into the CZTSSe lattice, and CNZTSSe thin films were obtained [22]. CNZTSSe thin films were used as an absorption layer to prepare CNZTSSe solar cells. The results indicate that the device properties and efficiency are significantly improved at the appropriate concentration of Ni doping. It is well known that selenidation conditions are very important for the grain growth of thin films [23,24,25]. To further obtain high-quality CNZTSSe devices, it is no doubt to optimize the selenidation conditions. Up to now, the effect of the selenidation time on the structural and photoelectric characteristics of CNZTSSe films, as well as the performance of devices, has not been reported.

This work is the first time selenizing CNZTS precursor films synthesized a series of CNZTSSe films at different selenidation times. The impacts of selenidation time on the crystallization, optical and electrical performances of CNZTSSe films were systematically studied. Meanwhile, the influence of selenidation time on the photovoltaic property of CNZTSSe devices was also studied by adjusting the selenidation time. Finally, CNZTSSe solar cells with a photoelectric conversion efficiency of 5% were obtained by optimizing the selenidation time.

## 2. Experimental Section

### 2.1. Materials

**Figure 1:**
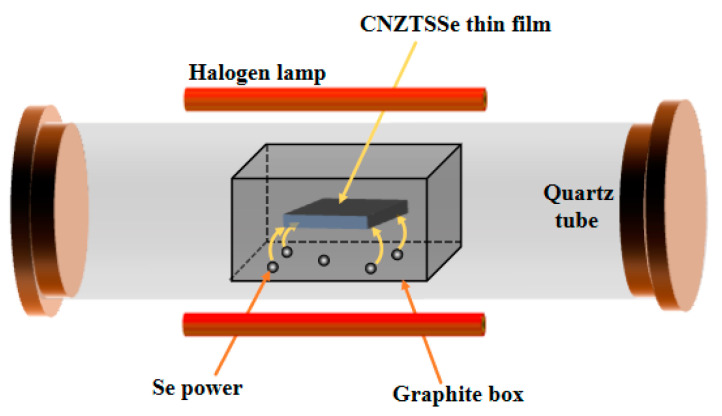
Schematics of graphite box used for selenization annealing.

The Cu_2_Ni_0.05_Zn_0.95_SnS_4_ (CNZTS) film was deposited on Mo deposited glass substrate (Mo-SLG) by sol-gel means. Firstly, the precursor solution of CZTS was obtained by dissolving 1.1979 g Cu(CH_3_COO)_2_·H_2_O (99.9%), 0.5693 g ZnCl_2_, 0.8462 g SnCl_2_·2H_2_O (99.9%), 0.0639g NiNO_3_·6H_2_O (99.9%) and 2.2836 g thiourea (99.9%) in 10 mL dimethyl sulfoxide. Ethanolamine (MEA) was selected as the stabilizer of the precursor solution. The doping concentration of Ni in the precursor solution is 0.05. The clear, light green solution was obtained by heating and stirring. CNZTS precursor solution was coated on Mo-SLG substrate at 3000 rpm for 30 s, then annealed at low temperature in a nitrogen atmosphere for 3 min to prepare CNZTS thin films. To meet the experimental requirements for the optimal thickness of 900 nm, it is necessary to repeat rotary coating and dry it ten times. Finally, the thin films were annealed in the graphite box using 0.02 mg Se powder (Figure 1). Figure 1 shows various components of a rapid thermal annealing furnace, including the halogen lamp, graphite box, and quartz tube. The selenization annealing treatment was divided into two steps. First, the temperature was raised to 200 °C after 80 s at room temperature, and then the temperature was raised to 540 °C after 135 s. The CZTSSe film was formed at this temperature for 900 s. The best CNZTSSe film can be obtained by adjusting the selenidation time under a selenidation temperature of 540 °C.

### 2.2. Fabrication

A 60 nm CdS buffer layer was synthesized on the surface of the CNZTSSe thin film absorption layer using the chemical bath deposition method. The 50 nm ZnO and 250 nm ITO window layers were synthesized on the CdS buffer layer by magnetron sputtering. The Ag top electrode was deposited on the ITO layer by evaporation technique. Thus, CNZTSSe devices with SLG/Mo/CNZTSSe (x = 0.05)/CdS/i-ZnO/ITO/Ag traditional structure were obtained.

### 2.3. Characterization

The crystal structure of CNZTSSe films was studied by X-ray diffraction (XRD) and Raman spectroscopy. The types and contents of elements in films were determined by X-ray photoelectron spectroscopy (XPS) and energy dispersive spectroscopy (EDS) systems. Using scanning electron microscopy (SEM) to characterize the morphology of CNZTSSe films. The optical and electrical performances of the films were characterized via ultraviolet-visible near infrared spectrophotometer (UV-3101pc, Tokyo, Japan) and Hall test. The photovoltaic performance of the device was characterized by the current density voltage (J-V) curve and external quantum efficiency (EQE) curve (zolix solar cell scoan100) test.

## 3. Results and Discussion

### 3.1. Effect of Selenidation Time on the Structure and Morphology of Thin Films

**Figure 2:**
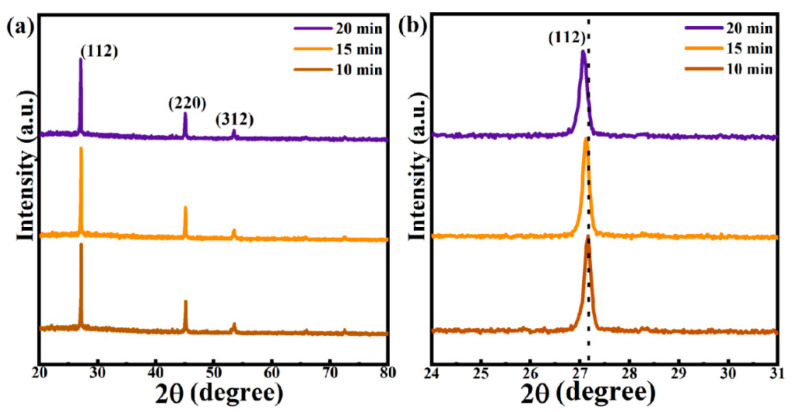
(**a**) XRD patterns of CNZTSSe films selenized at various selenidation times; (**b**) Enlarged images of (112) peaks of CNZTSSe films selenized at various selenidation times.

**Figure 3:**
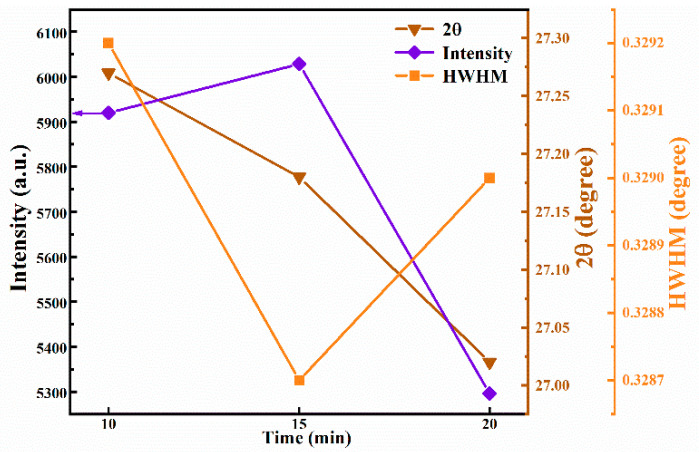
Curves of the 2θ, peak intensity, and FWHM of (112) peaks for the CNZTSSe films with the selenization time.

The precursor film was deposited on Mo-SLG substrate using CNZTS solution and annealed at low temperature. Then, the CNZTS film was selenized to form the CNZTSSe absorption layer film. The selenidation procedure is the same as preparing the CZTSSe films [26]. The selenidation temperature of CNZTS is 540 °C instead of a higher temperature to avoid decomposition [27]. Figure 2a exhibits the XRD patterns of CNZTSSe films prepared under different selenidation times. In the whole spectrum, the diffraction peaks at 27.06°, 45.04°, and 53.48° belong to the crystal plane diffraction peaks of the CZTSSe phase, and the corresponding crystal planes are (112), (220), and (312), respectively. No characteristic peaks of other impurity phases are observed [28]. It means that the pure phase CNZTSSe film was obtained. In addition, the peak intensity reached the maximum value when the selenidation time was 15 min. This confirmed that the crystallinity of the CNZTSSe phase was improved by adjusting the selenidation time. Figure 2b shows an enlarged image of the (112) peaks. It can be more clearly observed that the (112) peak position shifts to a small angle with the extension of selenidation time, indicating that the larger Se atom replaces the smaller S atom in CNZTSSe film. The half peak width (FWHM), peak position (2θ), and peak intensity of the (112) main peak are integrated into Figure 3.

According to the changing trend of the curve in Figure 3, it is intuitively observed that FWHM, 2θ, and peak intensity changed significantly. As the selenidation time increases from 10 min to 15 min, the corresponding FWHM decreases gradually, and the corresponding peak intensity gradually increases, which strongly demonstrates that the crystallinity of the film was improved. As the selenidation time reached 15 min, the peak intensity reached the maximum, and the FWHM was the narrowest. However, as the selenidation time was raised to 20 min, the peak intensity decreased significantly, and FWHM widened. This may be due to the increase in selenidation time and large holes on the film surface. In addition, the 2θ value gradually shifts to a smaller angle with the extension of selenidation time, which can be more intuitively observed in Figure 1. This can be attributed to the fact that the degree of substitution of Se increases with the selenidation time because the radius of the Se ion is greater than that of the S ion.

**Figure 4:**
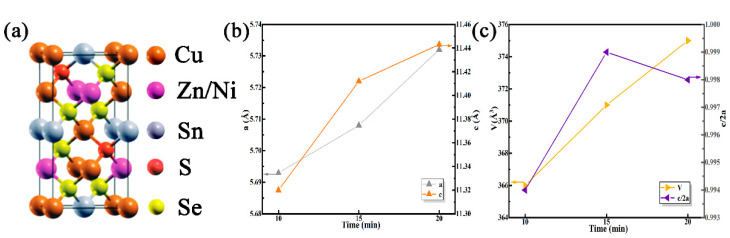
(**a**) Cell structure diagram of CNZTSSe film; (**b**) Lattice parameters a and c of CNZTSSe films obtained at the selenization time of 10, 15, and 20 min; (**c**) Unit cell volume (V) and η = c/2a of CNZTSSe thin films obtained at the selenization time of 10, 15 and 20 min.

To explore the impact of selenidation time on the structure of the film, the lattice constants a, c, and lattice volume (V) of the thin film were obtained by analyzing and calculating the XRD test results. The lattice parameters of the tetragonal structure can be calculated by this formula

$$\frac{1}{d_{\mathrm{hkl}}{}^{2}}=\frac{h^{2}{+k}^{2}}{a^{2}}+\frac{l^{2}}{c^{2}}$$

where d_hkl_ is the distance between (hkl) crystal planes. Figure 4a shows the lattice structure of CNZTSSe. It can be seen that Ni replaces the Zn position, and Se replaces the S position. Figure 4b,c shows that the lattice constants a and c gradually increased with the selenidation time, and the V of the corresponding films expanded. This further confirms that the larger Se replaces the smaller S. In addition, the corresponding η value of the film is obtained by calculating the c/2a. It can be found from Figure 4c that the values of η are less than 1. The structure study of Quaternary chalcogenide semiconductors displays that the η > 1 and η < 1 represent stannite and kesterite structures separately [29]. Therefore, it was proved that the CNZTSSe films prepared at different selenidation times have a kesterite structure.

**Figure 5:**
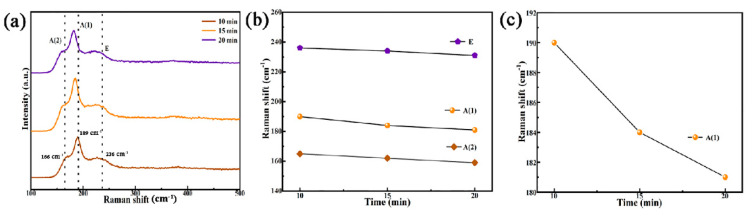
(**a**) Raman spectra of CNZTSSe films obtained at various selenization times; (**b**) Variation curve of various vibration mode peaks with selenization time; (**c**) Variation curve of main Raman vibration mode A (1) with selenization time.

Although the XRD patterns confirmed the formation of the kesterite CZTSSe phase, the peaks could not be distinguished between CZTSSe and secondary phases like Cu_x_S, cubic ZnS, and tetragonal Cu_2_SnS_3_ because of their similar lattice parameters [30]. To further analyze the purity of the film phase, the Raman test was carried out to distinguish between these secondary phases and kesterite CZTSSe. The Raman spectra of the films prepared at the selenidation time of 10, 15, and 20 min are shown in Figure 5a. The Raman spectra have three vibration peaks at 165, 189, and 235 cm^−1^, respectively, corresponding to A (2), A (1), and E vibration modes. The three vibration peaks originate from the CZTSSe phase with a kesterite structure [31]. No peaks of other impurity phases were detected in the spectrum, which confirmed that all CNZTSSe films have kesterite structures. In addition, the film with a selenidation time of 15 min showed a strong characteristic peak at 189 cm^−1^. Therefore, it was concluded that 15 min was the optimal selenidation time for forming pure phase CNZTSSe. Figure 5b shows the changes in peak positions of three vibration modes with selenidation time. Among three vibration peaks, the peak position of A (1) vibration mode changes significantly, which has a redshift with increased selenidation time (Figure 5c). This phenomenon is interpreted as that with the increase of selenidation time, the amount of Se atoms increases in the film, which may cause new interactions. So, the chemical bond vibration is weakened to make the Raman peak red shift [32].

**Figure 6:**
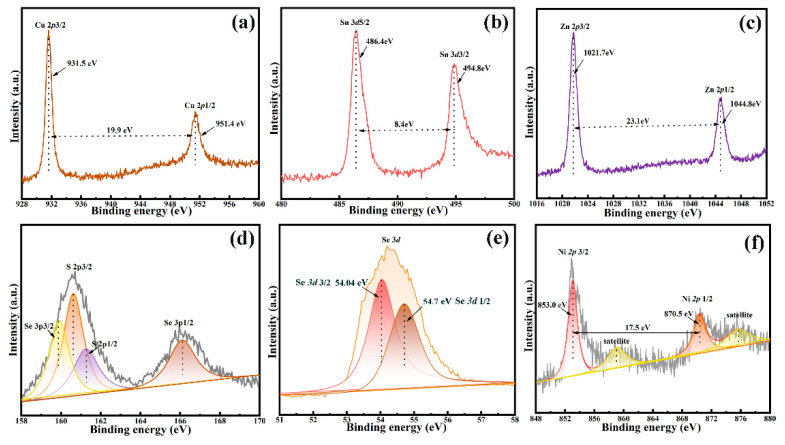
XPS spectra of CNZTSSe films selenized at 15 min: (**a**) Cu 2p, (**b**) Sn 3d, (**c**) Zn 2p, (**d**) S 2p, (**e**) Se 3d and (**f**) Ni 2p.

Figure 6 indicates the XPS spectra of all elements in the CNZTSSe films at the optimum selenidation time (15 min). Figure 6a displays the XPS spectrum of Cu 2p core energy level. The Cu 2p 3/2 and Cu 2p 1/2 peaks were obtained at 931.5 and 951.4 eV, and the peak position difference was 19.9 eV. This difference value is consistent with the binding energy of Cu^2+^, indicating that the Cu is + 2 valence in the film [33]. Figure 6b displays the XPS spectrum of Sn 3d core energy level. The two peaks of Sn 3d are located at 486.4 eV and 494.8 eV, with a binding energy difference of 8.4 eV. Therefore, the above two peaks are tetravalent Sn 3d 5/2 and Sn 3d 3/2 (Sn^+4^), separately [34]. Figure 6c displays the peaks of Zn 2p 3/2 and Zn 2p 1/2 at 1021.7 eV and 1044.8 eV. The peak level difference is 23.1 eV, which conforms to the binding energy of Zn^2+^, indicating that Zn exists at + 2 valence in the film [35]. The XPS spectrum of S 2p (Figure 6d) indicates the four peaks of S 2p 3/2, S 2p 1/2, Se 3p 3/2, and Se 3p 1/2, located at 160.3 eV, 161.2 eV, 159.7eV and 166.0eV, separately. The two peaks at 160.3 eV and 161.2 eV have a binding energy difference of 0.9 eV [36]. This agrees with the 160–164 eV range of S^2−^, revealing that the valence state of S is + 2 [36]. The Se 3d 3/2 and Se 3d 1/2 peaks are respectively located at 54.04 and 54.7 in Figure 6e. The split value is consistent with the binding energy value of Se^2−^ [37]. In Figure 6f, Ni 2p 3/2 and Ni 2p 1/2 core levels with a peak separation of 17.5 eV were observed at 852.9 and 870.4 eV, respectively, indicating that Ni atoms with a valence state of + 2 have been successfully doped into CZTSSe films [38]. In addition, there are two satellite vibration peaks in the XPS spectrum of the Ni 2p core level, which does not affect the results of Ni valence analysis [39]. XPS test displays that the constituent elements CNZTSSe film exist in the forms of Cu^+^, Zn^2+^, Sn^4+^, S^2−^, Se^2−^, and Ni^2+^.

The element contents in the films with different selenidation times were determined by EDS measurement. The test results are shown in Table 1. After selenidation treatment, the Cu/(Zn + Ni + Sn) and Zn + Ni/Sn ratio in the films are unchanged. The value of Ni/(Ni + Zn) in all films has little change, which reveals that the effect of selenidation time on Ni doping content is weak. The most obvious change is the negative correlation between S and Se elements in the film. It means that Se replaces more S in the lattice as the increase of selenidation time, which leads to a significant increase in the content of Se. The increase of Se content helps to reduce the band gap of the film, which will be proved later. In addition, it can be found from Table 1 that the Se + S content reaches 50%. It means that the film is in a selenium-rich environment at this time.

**Figure 7:**
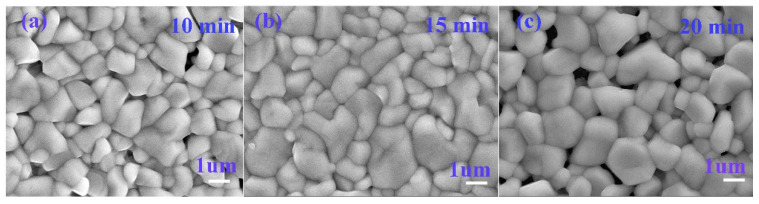
Surface morphology of the CNZTSSe thin films selenized at 10 min (**a**), 15 min (**b**), and 20 min (**c**).

Figure 7a–c displays the surface SEM images of CNZTSSe films prepared separately at selenidation times of 10, 15, and 20 min. It can be observed that the grain size of the film reaches the micron level from the nano level with the increase of selenidation time. When the selenidation time is 10 min, the surface grain of the film is small, the surface is rough, and there are many holes (Figure 7a). Figure 7b displays that the film is composed of large particles. It has a smooth and dense surface and almost no holes when the selenidation time reaches 15 min. However, when the selenidation time is further increased to 20 min, although the grain size of the film is large, some holes can be seen on its surface (Figure 7c), which is not conducive to carrier transport. The results show that the increase in the selenidation time can promote grain growth, and the optimal selenidation time is 15 min. A longer selenidation time may lead to the decomposition of the CNZTSSe phase and increase the pores on the surface of the film [40].

### 3.2. Effect of Selenidation Time on Optical and Electrical Properties of Thin Films

**Figure 8:**
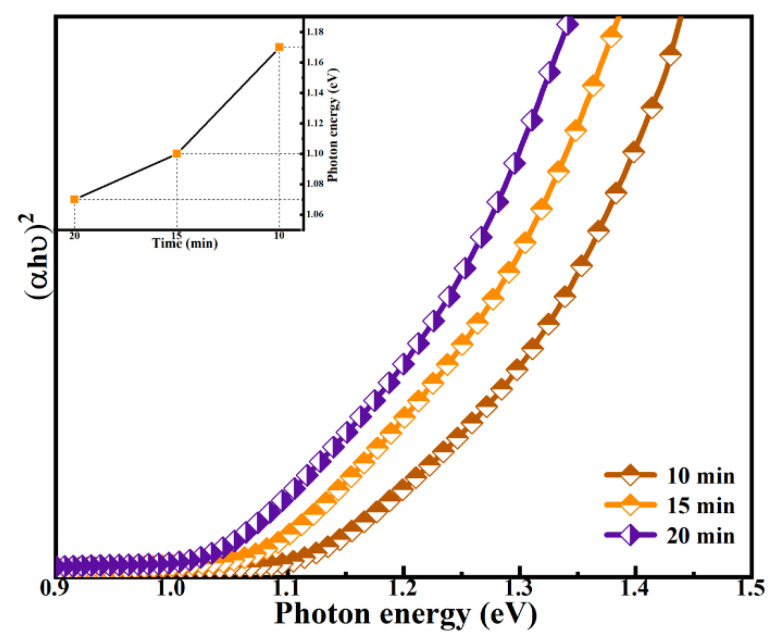
The curve of (αhυ)^2^ vs hυ for CNZTSSe films selenized at 10 min, 15 min, and 20 min; The insert shows the variation curve of Eg with selenidation time.

The curves of (αhυ)^2^ versus the photon energy (hυ) for the selenided films at the selenidation time of 10, 15, and 20 min were drawn in Figure 8. The absorption spectrum of the films was processed by using the Tuac’s relation (1), and the optical band gaps of the films were obtained.

(*αhυ*) = *B*(*hυ* − *E*_g_)^1/2^ (1)

The *α* is the optical absorption coefficient, *B* is the Planck constant, and *hυ* is the photon energy. The intercept between the tangent of the curve and the *x*-axis is the Eg value in Figure 8. The Eg values of all films are summarized in the illustration of Figure 8. The Eg values are estimated to be 1.17, 1.10, and 1.07 eV for the CNZTSSe films at the selenidation time of 10, 15, and 20 min, separately. As the increase of selenidation time, the substitution of Se for S in the film increases, resulting in a decrease in S content. As the decrease of S content, the scattering of electrons in the forbidden band at the grain boundary weakens, which will promote the transition of electrons in the forbidden band, thus reducing the Eg value [41]. On the other hand, according to the first principle calculation, the valence band maximum (VBM) of CZTS (CZTSe) is mainly related to S 3p (Se 4p) and Cu 3d orbital hybridization. The conduction band minimum (CBM) is mainly related to S 3p (Se 4p) and Sn 3d orbital hybridization [42]. However, when S is replaced by Se and Se content increases, the orbital interaction between CBM and VBM decreases, thereby reducing the Eg [43]. The results mean that adjusting selenidation time is an effective means to adjust the Eg of CNZTSSe films.

The electrical performances of CNZTSSe films were studied by Hall measurement, and the test results were recorded in Table 2. The Hall results demonstrated that irrespective of the dwell time during the selenidation process, all films exhibited p-type conductivity. As the increase of selenidation time from 10 to 15 min, the carrier concentration increased from 1.21 × 10^16^ to 1.68 × 10^16^ cm^–3^. Meanwhile, the mobility increased from 3.39 to 3.78 cm^2^V^−1^s^−1^, and the resistivity reduced from 9.98 × 10^2^ Ω·cm to 1.51 × 10^2^ Ω·cm. Then, when the selenidation time extended to 20 min, the carrier concentration decreased slightly to 1.66 × 10^16^ cm^–3^, the mobility decreased to 2.12 cm^2^V^−1^s^−1^, and the resistivity increased to 1.75 × 10^2^ Ω·cm (see Table 2). Owing to the increase of the crystallinity and the decrease of grain boundary in the film with the increase of selenidation time to 15 min, increasing carrier concentration and mobility and decreasing resistivity. As the selenidation time increased to 20 min, the quality of the film became worse, which was characterized by large holes, rough surfaces, and increased grain boundaries. This worsens the overall electrical performance of the film. Hence, based on the above analysis, the electrical performance of the film is the best at the selenidation time of 15 min, which meets the requirements of the ideal absorption layer of CNZTSSe solar cells.

### 3.3. Effect of Selenidation Time on Device Performance

**Figure 9:**
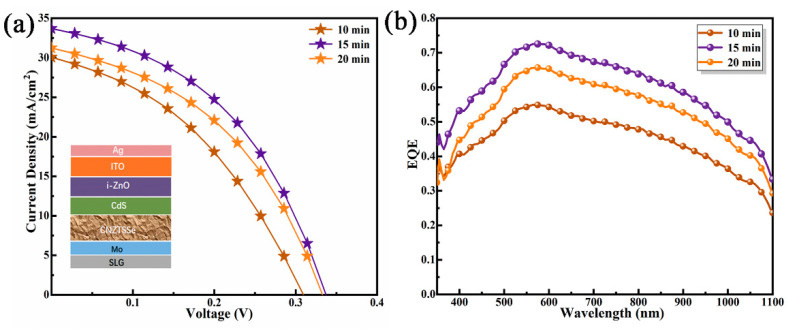
(**a**) J-V characteristic curves of CNZTSSe solar cells at the selenidation time of 10, 15, and 20 min. (**b**) Corresponding EQE curves of CNZTSSe solar cells.

**Figure 10:**
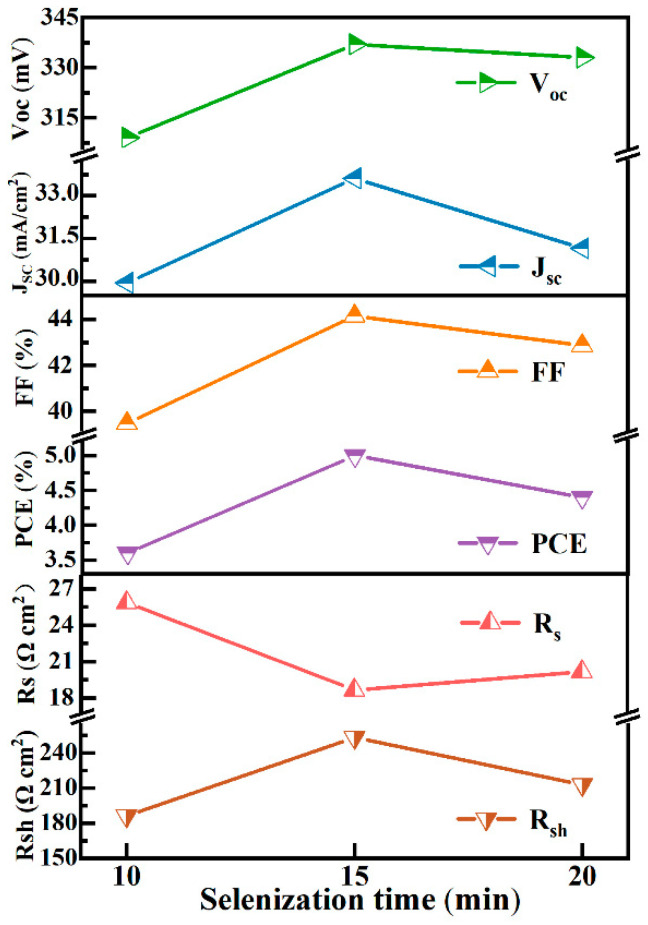
Variation curve of main device parameters as selenidation time.

CNZTSSe solar cells with a conventional structure were illustrated in Figure 9a inset. Figure 9a displays the J-V curves of the CNZTSSe solar cells. The main photovoltaic parameters were recorded in Table 3. It displays that the photoelectric conversion efficiency (PCE) increases from 3.6% to 5.0% as the increase of selenidation time from 10 to 15 min. As the selenidation time was raised to 20 min, PCE decreased to 4.4%. Moreover, it can be found that other device parameters of the three devices, such as V_oc_, filling factor (FF), short-circuit current density (J_sc_), and shunt resistance (R_Sh_), which have similar variation laws with PCE. They all increased sharply as the increase of selenidation time from 10 to 15 min and then decreased from 15 to 20 min. The change law of series resistance (R_S_) with selenidation time shows an opposite trend. The device has the best performance parameters when the selenidation time is 15 min. The V_oc_, J_sc,_ and FF are 337 mV, 33.61 mA/cm^2,^ and 44.15%. The corresponding R_s_ and R_sh_ are 1.86 Ωcm^2^ and 253.05 Ωcm^2^, respectively. The curve of the relevant device performance parameters of the devices is shown in Figure 10. When the selenidation time is in the range of 10–15 min, there is a decrease in R_S_ and an increase in R_Sh._ This is due to the enhancement of the electrical properties and quality of the device and the reduction of Sn_Zn_ and Cu_Zn_ defects by replacing Zn with Ni in the absorption layer [44]. The decrease in R_S_ and the increase in R_Sh_ will enhance FF and J_sc_ [45]. When the selenidation is 15 min, the CNZTSSe has the best crystal quality. The R_Sh_ reached a maximum, and R_S_ had a minimum. Meanwhile, the CNZTSSe device obtained the highest PCE. Therefore, it is concluded that the change of the PCE with selenidation time is mainly decided by the variation of R_S_ and R_Sh_, which is relevant to the defect density and crystal quality of CNZTSSe films.

Figure 9b displays the external quantum efficiency (EQE) of CNZTSSe solar cells at selenidation times of 10, 15, and 20 min, respectively. Comparing the EQE spectra of the three devices at short wavelengths (less than 500 nm), it can be found that the optical response is significantly enhanced for the device with a selenidation time of 15 min. This indicates that the absorption from the CdS/i-ZnO/ITO top layer may be improved [45]. When the EQE spectrum extends to a longer wavelength region, the optical response of the device increases as the increase of selenidation time from 10 to 15 min. For the device with a selenidation time of 15 min, the overall enhancement of the EQE spectrum can be attributed to the ideal crystal quality of the CNZTSSe film and less carrier recombination [46]. When the selenidation time further increases to 20 min, the optical response of the device decreases, which can be explained by the poor crystal quality of the absorption layer and the reduction of carrier transmission efficiency. The above analysis of EQE spectrums demonstrates that the CNZTSSe device with a selenidation time of 15 min performs best in collecting photogenerated carriers.

## 4. Conclusions

To summarize, we have synthesized a series of CNZTSSe thin films for the first time by selenizing CNZTS precursor films at different times, and successfully prepared CNZTSSe solar cells. Optimizing the selenidation time improved the performance of CNZTSSe films and devices. The results show that CNZTSSe film has high crystallinity, large grain size, and good electrical properties when the selenidation time is 15 min. In addition, the Eg of the CNZTSSe film is continuously adjustable with the change of selenidation time. As the selenidation time is 15 min, the PCE of the CNZTSSe device reaches the optimal value of 5%. The improvement of PCE is primarily due to the enhancement of crystal quality, the reduction of carrier recombination at grain boundaries, and good carrier collection ability. Based on the above research, optimizing the selenidation time of the absorption layer films provides a new idea for obtaining high-quality CNZTSSe solar cells.

## Figures and Tables

**Table 1:** EDS results of the CNZTSSe films at selenidation times of 10 min, 15 min, and 20 min.

Sample	Cu(% at)	Ni(% at)	Zn(% at)	Sn(% at)	S(% at)	Se(% at)	Cu/(Zn + Ni + Sn)	(Ni + Zn)/Sn
10 min	23.65	0.41	17.28	10.41	1.78	46.47	0.84	1.69
15 min	23.58	0.40	17.10	10.24	1.74	46.95	0.85	1.70
20 min	22.67	0.42	17.32	10.60	1.71	47.29	0.80	1.67

**Table 2:** The resistivity (Ω·cm), carrier concentration (cm^−3^), and mobility (cm^2^v^−1^s^−1^) of CNZTSSe films were obtained as the selenidation time of 10 min, 15 min, and 20 min.

Samples	Resistivity (Ω·cm)	Carrier Concentration (cm^−3^)	Mobility (cm^2^V^−1^s^−1^)	Type
10 min	1.51 × 10^2^	1.21 × 10^16^	3.39	p
15 min	9.98 × 10^1^	1.68 × 10^16^	3.78	p
20 min	1.75 × 10^2^	1.66 × 10^16^	2.12	p

**Table 3:** The main photovoltaic parameters of CNZTSSe solar cells were prepared at the selenidation time of 10, 15, and 20 min.

Device	Active Area	V_OC_ (mV)	J_SC_ (mA/cm^2^)	FF (%)	PCE (%)	R_s_ (Ω cm^2^)	R_sh_ (Ω cm^2^)
CNZTSSe (T = 10 min)	0.19 cm^2^	309	29.94	39.46	3.6	2.58	186.01
CNZTSSe (T = 15 min)	0.19 cm^2^	337	33.61	44.15	5.0	1.86	253.05
CNZTSSe (T = 20 min)	0.19 cm^2^	333	31.15	42.86	4.4	2.01	212.96

## Data Availability

Not applicable.
